# Developing a methodology to quantify mismanaged plastic waste entering the ocean in coastal countries

**DOI:** 10.1111/jiec.13349

**Published:** 2022-12-16

**Authors:** Diana Ita-Nagy, Ian Vázquez-Rowe, Ramzy Kahhat

**Affiliations:** Peruvian Life Cycle Assessment and Industrial Ecology Network (PELCAN), Department of Engineering, Pontificia Universidad Católica del Perú (PUCP), Avenida Universitaria 1801, San Miguel Lima, 15088 Peru

**Keywords:** industrial ecology, marine litter, Pacific Ocean, Peru, river basin, waste management

## Abstract

Marine plastic accumulation has gained international attention in recent years. Sources, pathways, and environmental impacts are being currently studied to understand the complex interactions during waste, especially plastic, transportation to the sea. Rivers have been identified as debris corridors allowing transportation of mismanaged waste. However, there is also evidence of waste accumulation in river basins, suggesting they can also act as sinks. Thus, assuming a uniform and continuous transportation of waste through rivers towards the ocean may signify an oversimplification. This study proposes a methodology to estimate plastic release to the ocean, considering a more detailed characterization of each river basin, including natural attributes and manmade constructions that may act as barriers or boosters for this release. The methodology is exemplified using a case study for the Region of Piura, Peru, and estimating a range of 4.2 to 13.9 kg/person/year of plastic waste reaching the Pacific coast during 2018. These results, when compared with the existing literature, demonstrate more conservative estimations. This methodology is presented as a useful tool that can be easily applied to develop more accurate mismanaged waste dissipation along different compartments.

**Supplementary Information:**

The online version of this article (doi:10.1111/jiec.13349) contains supplementary material, which is available to authorized users.

## INTRODUCTION

Increasing awareness regarding the accumulation of litter in the oceans, especially plastic waste, has fostered a surge in studies that analyze their environmental impacts (Lavoie et al., 2021) and the pathways through which residues are released to the ocean (Woods et al., 2021). Current research suggests that a substantial fraction of ocean waste is linked to terrestrial-based activities and mismanagement of waste in coastal cities (Eunomia, 2016; GESAMP, 2016). However, geographical differences exist, mainly between developed countries and emerging and developing countries (Jambeck et al., 2015). While releases in Europe and the United States are mainly related to tire abrasion, microplastics in wastewater, or coastal littering, in the Global South the absence of adequate integrated municipal solid waste (MSW) treatment systems is the main carrying factor (Napper & Thompson, 2018). In fact, Margallo et al. (2019) highlighted that although most MSW (above 90%) in Latin America and the Caribbean is correctly collected, it fails to be disposed of using adequate waste treatment procedures. This leads to a large proportion of waste being sent to open dumpsters or mismanaged landfills (Ziegler-Rodriguez et al., 2019). Additionally, considering that accelerated growth of urban areas in developing countries leads population to settle, most of the time informally, in unsafe locations (e.g., river floodplains) where the common practice for solid waste disposal are open dumps, the risk of litter reaching water bodies increases (Blettler et al., 2018).

There is a strong causality between increasing concentration of marine litter and cities, especially when located in coastal areas or close to rivers (Meijer et al., 2021). This correlation increases with deficient waste collection systems (Roebroek et al., 2021). Two studies conducted in the past decade estimated the amounts of solid waste, specifically plastic, that are transported by rivers or coastal cities to the sea (Lebreton et al., 2017; Schmidt et al., 2017), identifying rivers as massive discharge agents. However, there is also evidence of long-term waste accumulation in river basins (e.g., riverbanks, fluvisols), suggesting that rivers actually present a dichotomous behavior, in which they can also act as sinks. This is the case in areas located farther away from the coast, but relatively close to a basin's catchment area (Winton et al., 2020). Thus, rivers are a crucial actor for marine litter, functioning as connectors between populations, mismanaged waste, and waste flows to the sea (Meijer et al., 2021).

To date, studies that quantify the waste flow entering the ocean have been performed at a large scale considering the capacity of nations to manage their solid waste, their population (Jambeck et al., 2015) and, in some cases, information related to river hydrology (Lebreton et al., 2017). However, we argue that a set of natural- and human-based barriers exist, as well as other carrying factors, such as river morphology and size, wind speeds, or agricultural drainage systems that play an important role in the final release of waste to the ocean or, conversely, contribute to the accumulation of this waste in terrestrial sediments. Hence, the retention of solid waste, namely plastic, at a watershed scale has remained largely excluded in the methodological evaluation of global or regional solid waste releases to oceans. Moreover, most studies related to the evaluation of litter mobilized through rivers are performed in developed countries, generating the need to increase the assessment in the Global South (Lahens et al., 2018).

Therefore, the aim of this study is to describe the driving factors affecting the transport and fate of solid waste throughout a basin in the Global South, using the Peruvian Pacific basin as the geographical area of reference, and their relationship with marine plastic litter. An important amount of Peruvian MSW (ca. 40%) ends up in open dumpsters, as a consequence of a poor waste management system (MINAM, 2021). These dumpsites are not only located in open areas, but also close to riverbanks or at the seashore. The lack of controlled waste disposal management sites throughout the main cities, except Lima, along the Peruvian coast (e.g., Arequipa, Trujillo, Chiclayo, or Piura) (Cristóbal et al., 2022), as well as the main tourist destinations, suggests that important releases of waste, including plastics, are reaching the Pacific Ocean. After this evaluation, a more specific quantification scheme to estimate the amount of plastic waste that is either released or retained at a watershed scale can be developed for a more accurate estimation of the mismanaged plastic waste that could be arriving at the ocean. Consequently, we are proposing a novel methodology considering the different natural or artificial barriers that mismanaged plastic waste encounters until reaching the ocean, in order to re-evaluate the way transport and fate of waste has been previously assessed from terrestrial sources. The methodology is presented to exemplify the effects on the final amount of plastics reaching the ocean from inland sources. The case study is conducted to illustrate the relevance of the effects of retention and release of materials caused by the different barriers identified. Further work, thus, is needed in order to refine the factors given in this study.

## THE EFFECT OF NATURAL AND ARTIFICIAL BARRIERS ON WASTE TRANSPORTATION

Plastic waste reaches the ocean through a wide array of routes, including intentional littering in coastal areas or river basins, especially in areas with poor waste management, and accidental losses caused by natural events like intense rainfalls, tsunamis (Murray et al., 2018), or strong winds (Schöneich-Argent et al., 2019). To estimate marine litter accumulation, different parameters can be used, such as mismanaged waste generation (Ita-Nagy et al., 2021), population size and density near coastal or river watersheds (Schmidt et al., 2017), distribution of urban infrastructure (van Emmerik et al., 2019), amongst others. However, natural and anthropogenic barriers during waste transportation should also be identified, as they play a determinant role in the final release to oceans. Flowing water bodies, namely rivers, show a critical role in the redistribution of debris in a basin (Meijer et al., 2021). The high variability in orography, industrialization, urbanization or population density of areas analyzed, therefore, greatly influences the final amounts of debris reaching the final sections of rivers and, ultimately, the ocean. This study proposes a river basin-specific method to estimate the amount of mismanaged MSW, with a focus on plastics, transported to the sea or otherwise retained in non-marine compartments.

Are natural and anthropogenic barriers responsible for preventing part of the mismanaged solid waste fraction from reaching the sea? In previous studies that analyze solid waste routes to the sea, rivers are mentioned as the main transport pathways (Jambeck et al., 2015; Roebroek et al., 2021). However, the obstacles or barriers that waste encounters during this trajectory are not mentioned, at least not directly linked to waste flows. Rivers are not uniform in terms of morphology, length, width, turbulence, or flow, and may also have different natural or artificial barriers or sinks along their course. These barriers may partially or totally prevent suspended or floating waste debris from travelling downstream to the sea (Winton et al., 2020). Moreover, river morphology and size may play an important role when analyzing the transport and fate of litter (Lebreton et al., 2017). Thus, assuming a uniform behavior and transportation of waste in rivers, including their final destination, may constitute an oversimplification of the system that could provide erroneous estimations.

Hence, we propose a more detailed characterization for river basins to classify these according to the attributes of their main river (e.g., flows, seasonality), as well as tributaries, and the natural and/or artificial barriers along their route (e.g., hydroelectric plants). For this, we analyzed the Peruvian Pacific basin, which includes 53 river basins. The selection of this geographical area is justified by the characteristics of the Peruvian coast which, despite general hyper-arid conditions, presents a high level of variability between basins, including differences in river morphology, presence of hydropower infrastructure, local or regional integrated waste management systems, among others. Thereafter, the methodology has been applied to the Region of Piura (NW Peru) as a case study, although it can easily be applied to other geographic conditions.

## METHODOLOGICAL FRAMEWORK

The model formulation started with field work to observe and understand MSW behavior. Intensive research discussion took place, together with on-site identification of sinks, barriers, and boosters during MSW riverine transportation. Thereafter, it was evident that different manmade and natural barriers play a determinant role in waste transportation. Thus, the most relevant barriers were identified and described, allowing the development of a model to quantify the final amount of plastic waste-to-ocean (pWtO), after going through several barriers.

The methodological framework characterizes the basin and then quantifies the plastic waste that enters the ocean. It is based on two main mechanisms: (i) observation through field studies; and (ii) data gathering related to the basins assessed. Bibliographical data disclosed by different Peruvian institutions, as shown in Table Table 1, permitted mapping the entire Peruvian coastal basin considering key aspects (e.g., landfills, dumpsters, or manmade infrastructure).

**TABLE 1:** Sources of information included in the assessment of mismanaged municipal solid waste along the Peruvian Pacific basin

Type of information	Reference
Waste generation per capita per district, plastic fractions	SIGERSOL (2018)
Population per district	INEI (2017)
Recycling rates	MEF and MINAM (2020)
Sanitary landfills	MINAM (2021)
Open dumpsters	OEFA (2021)
Hydroelectric plants	OSINERGMIN (2020)
Dams, irrigation infrastructure	ANA (2020)

When data availability and quality were lacking, assumptions were considered. For instance, MSW generation rate per capita was not available for ca. 14% of the population in some municipalities, and therefore, weighted averages were calculated instead (see Supporting Information Table S1 of Supporting Information S2 for more details). Moreover, in the case of material recovery, by waste segregation, official numbers were used. However, during field trips, it was observed that informal waste pickers recuperate hard plastics, cardboard, metal, and other materials, segregated in open dump premises. Thus, the effect of informal recyclers along the transport path of plastic waste was considered. However, important limitations exist when estimating this fraction (see Section 3.1).

In parallel, bibliographical data were complemented with field work performed in 2020 and 2021. Table S2 (in Supporting Information S1) describes the field trips performed. Activities during field work included the observation of river morphodynamics, agriculture drainage systems, the urban water cycle in major cities, open dumpsters and landfills, and the dynamics occurring at river mouths and beach shores. Although the case study only covers the Region of Piura, field work covered a larger area to have a clear understanding of the pathway mismanaged MSW may follow under different basin and area characteristics. Moreover, data were also collected from previous studies that analyzed watersheds (Salmoral et al., 2020), open dumpsters and landfills (Ziegler-Rodriguez et al., 2019), hydropower infrastructure in the Peruvian Andes (Verán-Leigh & Vázquez-Rowe, 2019), water availability and infrastructure in major cities (Torre et al., 2021; Vázquez-Rowe et al., 2017), and the release of urban debris due to natural disasters (Garcia-Torres et al., 2017; Mesta et al., 2019, Parodi et al., 2021).

The combination of these efforts allowed obtaining a complete diagnosis of an important fraction of the Peruvian Pacific basin, identifying a set of natural- and anthropogenic-related features that can either boost or hinder the ability of water bodies, mainly rivers, to release anthropogenic waste into the ocean. Section 3.1 is devoted to describing and analyzing these main boosters or barriers that may influence these pathways. Section 3.2 presents an equation which includes riverine inputs as well as coastal direct inputs, as a first step to model the fate of macroplastics into the marine environment from inland sources. The coefficients proposed intend to illustrate the effects of natural or manmade barriers in the transport of solid waste in riverine systems. Further research considering the amount of waste retention, together with possible fragmentation and degradation mechanisms, should be performed to reduce the uncertainty of these coefficients.

### Step 1: Identification of natural and anthropogenic barriers

#### Urbanization and waste management systems

Urbanization in emerging and developing nations can be rapid, chaotic, and disorganized, creating communities with an absence of adequate infrastructure. Municipal authorities usually struggle to provide sufficient city-wide MSW (Margallo et al., 2019) and water and wastewater (Torre et al., 2021) treatment infrastructure. In Peru, most cities along the Pacific basin, except Lima and Callao, lack adequate disposition sites (i.e., sanitary landfills), with most of the MSW finalizing in open dumpsters or riverbanks (OEFA, 2018). Efforts to close the infrastructure gap have commenced, but still do not reach important cities (MINAM, 2021; Vázquez-Rowe et al., 2019). In smaller towns, open dumpsters are predominant, most of which lack daily or intermediate coverings, making waste debris prone to entering water bodies or soil sediments.

In terms of water infrastructure, the Peruvian network is mainly dense in urban areas, but lacks adequate maintenance (El Comercio, 2019). Approximately 82% of urban population is provided with potable water (VIVIENDA, 2021), which means that an important amount of water is channeled into the urban water cycle, generating a crucial barrier that separates solid waste and suspended solids, some of which may be larger microplastics. If this fraction is managed and disposed of correctly in landfills, its return to the natural environment is improbable. In fact, we hypothesize that implementing adequate water and wastewater treatment plants in the Andean water tower may benefit solid waste recovery from communities in the highlands and mid altitudes thanks to the terraced distribution of the population, mitigating the arrival of waste at the coastal floodplains or at the ocean.

Unfortunately, however, wastewater treatment in Peru is still a major weakness. Even in Lima, where coverage is high, treatment systems present low levels of sophistication (Torre et al., 2021; Vázquez-Rowe et al., 2017). Hence, Lima lacks the capacity to eliminate suspended particles below 1 mm in wastewater flows, leading to a substantial flow of micro- and nanoplastics into the ocean through outfalls. The situation in the rest of the country, except for few recently built wastewater treatment plants, is significantly worse, with most wastewater flows receiving no treatment (OEFA, 2014; Torre et al., 2021).

#### Characteristics of the river basins

River basins show variable characteristics throughout the upper, middle, and lower sections of the basin: morphology, related to the topography of their location, geology and groundwater conditions, vegetation within the drainage area or climate, including rainfall, humidity, and evaporation (Horton, 1932; Mahala, 2020). The interactions of the different characteristics define the behavior of each watershed, and directly affect their runoff (Liu et al., 2019).

**FIGURE 1:**
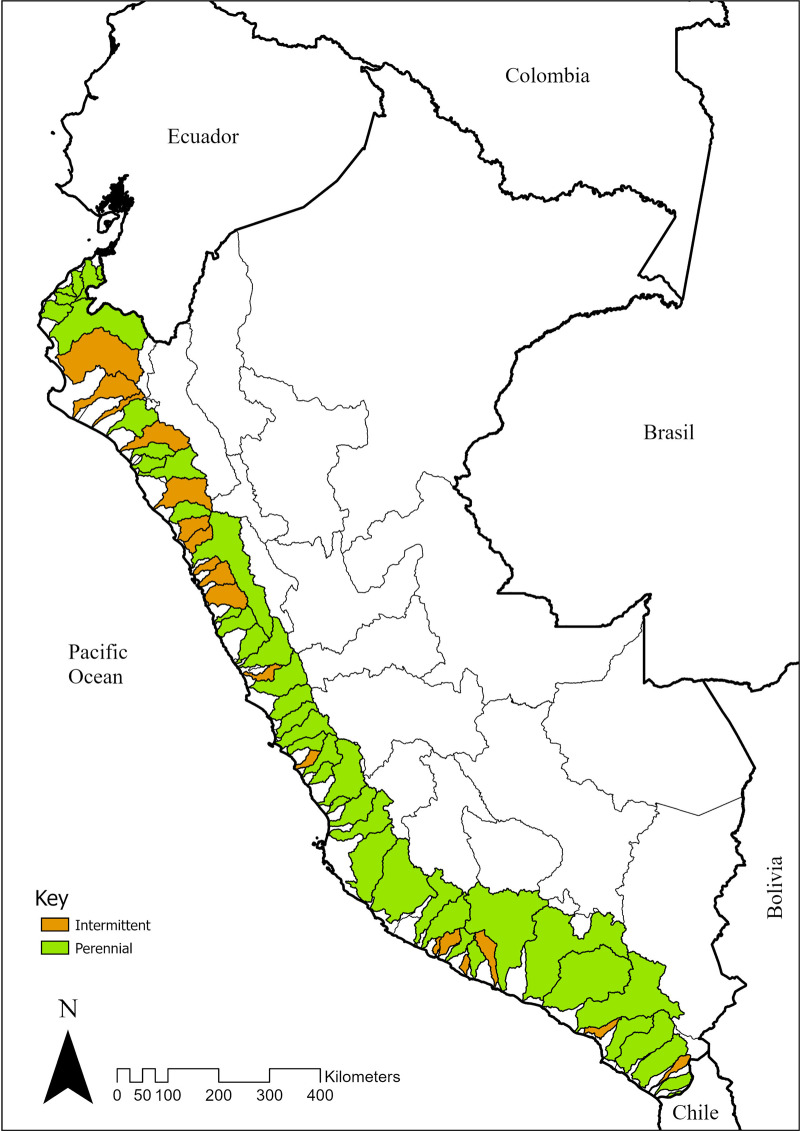
Classification of river draining basins in the Peruvian Pacific basin according to their seasonality. Green shade represents perennial basins, while orange indicates intermittent basins. Sixty-two (62) rivers make up this basin, 18 dry up during the dry season. The areas between basins, in white, are called inter-basins. Detailed information is available in Table S3 in Supporting Information S1

Along the Peruvian coast a succession of perennial and intermittent rivers can be observed (see Figure 1), depending on source location. Seasonality in the coastal strip is affected by relatively low rainfall, decreasing with latitude following a north-to-south gradient (Sanabria et al., 2018). For instance, the source of the river Rimac that flows through Lima is located in the heights of the Andes, providing a perennial flow of water from either rainfall or glacier melting. In contrast, the river Piura is located at lower heights, receiving substantially lower amounts of water in its catchment area, a situation that is partially regulated by the construction of hydraulic systems with water transfer from nearby rivers (AACHCHP, 2005).

This dichotomous trait of watersheds leads to different behaviors linked to the effective downstream transport of solid waste. Perennial rivers show rapid transport of solid waste in the upper basin, mixed with eroded materials and vegetation. If not subject to natural or anthropogenic barriers, these high gradient stream rivers can act as important sources of marine litter, especially in high density areas with abundant mismanaged MSW. Intermittent rivers lack a continuous flow to the ocean. In many cases they wash huge amounts of waste in short periods of time, but if they overflow riverbanks and floodplains, it may constitute an important sink for waste (Hurley & Nizzetto, 2018).

When plastic waste enters the water bodies in a given watershed from the technosphere or from other environmental compartments (e.g., aerial transport or reflux from the ocean), the size and shape of plastic particles will highly condition their mobility (Alsina et al., 2020; Besseling et al., 2017). In terms of size, it is plausible to assume that large macroplastic fragments will eventually be retained by anthropogenic barriers (e.g., dams), although natural disasters, as discussed below, could cause the displacement of large fragments, including construction materials or machinery. The fate of nano- and microplastics can be more challenging to track, since their mobility is affected by a wide range of processes, including homo- and hetero-aggregation (Wang et al., 2021), sedimentation–resuspension (Kukulka et al., 2012), polymer degradation (Chamas et al., 2020), presence of biofilm (Kaiser et al., 2017), and burial (Pohl et al., 2020). In fact, a study by Besseling et al. (2017) modeled their behavior, suggesting that nanoplastics and larger microplastics were more prone to be retained within the limnic ecosystem, whereas intermediate particles were likely to be washed downstream to the ocean. Shape has also been shown to have an influence on the sinking velocity of the particle, where round and smoother particles may experience higher settling velocity, when compared to irregular shaped ones (Khatmullina & Isachenko, 2017; Kowalski et al., 2016).

At the river mouth not all waste is transported offshore, but a percentage is washed ashore and is retained along the coast. However, there is still a lack of knowledge regarding the total waste reaching the ocean that is actually washed ashore (GESAMP, 2016). Wolf et al. (2020) have made progress by developing a machine learning system capable of quantifying washed ashore plastic items, which may be used to contribute to monitoring strategies in the future. Similarly, Collins and Hermes (2019), analyzed the different pathways and accumulation of microplastics in South Africa, estimating that 30% to 60% of those released are washed back to the coast.

#### Hydroelectric power plants

Hydroelectric plants were also identified as crucial manmade barriers in the Andean water tower. Hydropower is the main source of electricity in Peru (ca. 60% in 2020) (COES, 2021). Consequently, many Peruvian rivers that flow into the Pacific host numerous hydropower plants with sand and waste separation infrastructure. Most are run-off-river (ROR) plants, so not all the river flow is passed through sand or waste separation infrastructure. These infrastructures often include the employment of dams to control the intermittency of river flows (Verán-Leigh & Vázquez-Rowe, 2019). Additionally, hydropower stations are also coupled with screens located in the intake to remove suspended materials, solid waste, sand particles, and other debris (e.g., tree logs, plastic bottles) to avoid damaging the machinery. These screens are cleaned manually or mechanically, and removed waste is collected, entering the solid waste disposition cycle. A removal of 84% of plastic waste in hydroelectric plants was estimated in Bavaria (Witzig et al., 2021). However, considering that the final destination of the removed waste was unknown, this value was considered in the lower scenario (80% in this study) and other scenarios were included. Still, sand and other materials, in which nano- and microplastics may have hetero-aggregated, are returned into the riverbed through a flushing mechanism (Morris, 2020). Buoyant particles will continue their downstream flow, whereas others may be trapped in sediments (Klein et al., 2015). Unfortunately, as far as we were able to ascertain, studies to sample nano- and microplastic concentration in Andean fluvial sediments are yet to be conducted.

In upstream locations in the Peruvian Andes many villages and towns do not have wastewater treatment plants, making these plants an indirect basic form of wastewater treatment (Verán-Leigh & Vázquez-Rowe, 2019). Overall, it can be assumed that hydropower plants retain part of the mismanaged MSW, especially large elements that initiated their journey upstream (EUREC, 2019).

#### Irrigation canals and drainage systems

Irrigation channels and drainage systems for agriculture are common in coastal Peru (Damonte & Boelens, 2019). Most systems are composed of dams, main and secondary canals, sluice gates, and sand traps that behave as barriers or solid waste sinks. In agricultural and/or livestock production areas, parts of the rivers are channeled and re-derived into canals for irrigation. Hence, an important portion of the rivers’ water volume is concentrated for irrigation, and is infiltrated, absorbed by agricultural crops or evaporated, no longer working as a pathway for waste release into the ocean. However, in some cases, part of the channeled water will be discharged back into the riverine system, or into the ocean, possibly transporting solid waste added during their journey.

In contrast, we expect different processes to be enhanced. First, the accumulation of litter in agricultural fields may increase, depending on the concentration and type of solid waste disposed of upstream. This accumulation may affect soil quality, by, for example, increasing the accumulation of heavy metals (Ratul et al., 2018). Additionally, the accumulation of non-degradable litter, including plastics and microplastics, may affect the survival of terrestrial fauna (Lwanga et al., 2016).

#### Formal recycling and waste pickers

In developing economies, scavengers are key actors in the revalorization of recyclable waste (Wilson et al., 2006). Formal recycling, in contrast, represents a smaller fraction of total material recuperated. In Peru, it has been estimated that around 2% of all waste gets reinserted to the value chain and recycled (MEF & MINAM, 2020). Although waste pickers tend to be present anywhere waste accumulates (e.g., dumpsters, streets, river mouths, shorelines), quantifying the amount of waste recuperated by scavengers remains challenging (Sasaki & Araki, 2014), as the recuperation and sale of materials occurs mainly in the informal market (Wilson et al., 2006). Nonetheless, waste picking activities will affect the final amount of mismanaged waste entering the ocean; therefore, despite lack of adequate information and high uncertainty related to location, amount, and type of waste collected, their influence on waste recuperation should be included.

#### Wind speed and direction

Wind force has shown to be responsible for the transportation of loose solid waste, namely microplastics to the ocean (Prevenios et al., 2018), other water bodies (Eerkes-Medrano et al., 2015), or remote terrestrial areas (Allen et al., 2019). Shorelines oriented towards the Subtropical Convergence Zone and trade winds may show higher debris accumulation (Blickley et al., 2016). Considering the opposite situation, where coast lines are rarely affected by strong winds and heavy rain (e.g., the Peruvian coast), this absence could function as a barrier that limits the movement of litter towards the ocean. The Wind Atlas of Peru (MEM, 2016) shows the absence of strong winds throughout most of the coast, except some low-density populated areas in the regions of Ica (Nazca desert) and Piura (Sechura desert).

It is feasible to assume that mismanaged waste and littering located near the coastline or other waterbodies (i.e., rivers) in areas with stronger winds may be more prone to end in the ocean. However, although most open dumpsters in the region of interest lack coverings, it is plausible to assume that wind in most cases is not a carrying factor influencing the arrival of macroplastic waste at the ocean. In other words, the predominant light-to-moderate winds along the Peruvian coast (Correa et al., 2020; ENFEN, 2015) may only be responsible for the transportation of relatively light items, like plastic bags, which will still experience other types of barriers and retention (see Supporting Information S1, Figure S1). Having said this, it should be noted that tire abrasion, among other activities, are still responsible for important amounts of microplastic particles that are transported aerially into water bodies, especially since in some areas the Pan-American Highway is very close to the coastline (Verán-Leigh et al., 2019).

#### Natural disasters

The Peruvian coast is subject to recurrent natural disasters. Some are semi-cyclical, such as El Niño-Southern Oscillation (ENSO) (Cai et al., 2020). When an extreme ENSO event occurs, meteorology is affected, with a significant raise in the sea surface temperature, affecting rainfall, stressing watersheds, raising the amount of water transported by rivers, and, finally creating floods and landslides (Guzman et al., 2020). These dynamics potentially increase the amount of mismanaged MSW washed and disaster-related waste from areas located close to water bodies (Woods et al., 2021).

Past ENSO events have shown to be catastrophic in Peru, with significant human and economic loss (Parodi et al., 2021). Despite this phenomenon being deeply studied (Cai et al., 2020), some effects along the Peruvian coastline, for example, coastline variations caused by sediment transportation (Guzman et al., 2020), or mismanaged waste transportation, have been less evaluated. Although the negative effects in ocean and river contamination are easily observable after an extreme event (van Emmerik & Schwarz, 2020), most studies related to disasters are inclined to assess the economic and social damage (Parodi et al., 2021), with little evaluation of environmental consequences, including the probable increase of ocean waste. Considering that ENSO is likely to occur more frequently with more devastating consequences than in previous events due to climate change (Wang et al., 2019), augmenting the cascade effects it triggers in Peru and throughout the entire globe (Chen et al., 2017), we hypothesize that it will play an increasingly important role in the redistribution of marine litter.

Differently from waste driven through water bodies into the ocean along a regular year (mostly small- and medium-sized items), extreme events are responsible for transporting also larger fragments (e.g., construction waste, machinery, and debris), that otherwise are unlikely to end up in the ocean. Thus, areas at high risk should adopt strategies to diminish the effects of these events, including improved management of otherwise mismanaged waste, and strategies to prevent the washing of waste. Improving waste management in coastal and floodplain areas is an important strategy to consider, including better allocation of collection sites. However, there should be also a better understanding of the relationship between the occurrence of an extreme event and the subsequent augmentation of solid waste (Axelsson & van Sebille, 2017). In fact, a study conducted in England after an event of severe flooding demonstrated that a 70% reduction in the amount of microplastics in riverbeds may be attained due to flushing towards the river catchment (Hurley & Nizzetto, 2018), implying that these particles are being deposited in floodplains rather than washed to the ocean.

Beyond climate-driven events, Peru is also subject to significant seismic disturbance, with recurrent high intensity earthquakes and tsunamis (Yamazaki et al., 2010). Although certain studies have quantified the amount of urban waste that would be generated in Peruvian cities due to seismic events (García-Torres et al., 2017; Mesta et al., 2019), these are yet to be analyzed from a perspective in which leakage pathways to environmental compartments are evaluated. A study that evaluated the consequences of the earthquake and tsunami that struck Asia in December 2004 (Srinivas & Nakagawa, 2008), described and quantified how, during this event, different types of litter, including vegetation, MSW, or concrete accumulated in a close-by landfill site, were backwashed and transported into the ocean by the tsunami wave. Similarly, the Fukushima earthquake and the tsunami in 2011 were estimated to have washed 5 million metric tons of debris into the Pacific Ocean (Murray et al., 2018).

### Step 2: Measuring mismanaged plastic waste arriving at the ocean

Terrestrial sources have been found to be the major contributors to marine litter generation, especially marine plastics. Previous studies have provided efforts to quantify the amount of plastic waste that may be entering the ocean globally. The estimation by Jambeck et al. (2015) considered the total amount of plastic waste generated by 192 coastal nations in 2010, by correlating waste generation, population density, economic status, and closeness to the coast (first 50 km only). However, the estimate did not include the influence of rivers during waste transportation. Lebreton et al. (2017) followed a similar perspective by limiting the scope to the first 50 km; however, they estimated the waste input conducted by rivers including the effects of the river run-off and the presence of dams as an artificial barrier. Schmidt et al. (2017) published a similar study estimating waste input from the riverine system; however, artificial barriers were not included. Both studies developed models that were calibrated with macro- and microplastic sampling data from previous studies, including only plastic mobility in the water column, while excluding the presence of plastics in sediments. Finally, Meijer et al. (2021) performed a more refined estimation of waste entering from rivers with more precise data related to plastic waste generation, as well as climatological and geographical factors.

The current study seeks to provide an incremental update of these efforts and a more comprehensive approach, considering additional factors that should be included when quantifying marine litter, such as the direct input of coastline population, population located in inter-basins, and the effects of natural and artificial barriers in the riverine system. While these previous estimations can be considered fairly conservative, our work refines these calculations, and provides a more holistic equation to quantify the amount of plastics that enter the ocean.

Our model seeks to estimate the amount of the plastic content from MSW inputs into the ocean (i.e., pWtO). We define pWtO as the amount of plastic waste from MSW that is not disposed of in an adequate landfill or otherwise correctly treated through more sophisticated technology (Jambeck et al., 2015), and is likely to end up in the ocean. Plastic waste generated through littering in seashore activities (e.g., tourism) were excluded from the system boundaries. Having said this, it should be noted that to conduct a holistic assessment, littering and other types of plastic waste mismanagement must be included. However, the current study focuses on the importance of improving the metrics to report the release of pWtO from mismanaged disposition sites.

The equation proposed (see Equation 1) considers the most crucial barriers discussed above, as possible natural or anthropogenic sinks. Each barrier, depicted by the different factors described in the equation, may be capable of reducing or intercepting a fraction of the plastic waste in its way through each river basin and, thereafter, to the ocean. The estimation of total pWtO can be calculated per urban agglomerations, watershed, or as a whole country by adding up the values of each basin along the coast. Equation (1) is described as follows:

1 $$ \rm{pWtO}=\sum {Q}_{\rm{mMSW}{p}_{\textit{ij}}}\ast \left(1-{f}_{{\rm{sr}}_{i}}\right)\ast \left\{ \def\eqcellsep{\;}\begin{array}{cc}{{f}_{{\rm{cl}}_{i}},}\; \rm{if}0\;lt;\bar{x}\;lt;0.1\rm{km}\\ {{f}_{{\rm{ci}}_{i}},}\; \rm{if}\bar{x}\;gt;0.1\mathrm{km}\mathrm{in}\mathrm{an}\mathrm{inter}-\rm{basin}\\ {{f}_{{\rm{cw}}_{i}}\ast {f}_{{\rm{rs}}_{j}}\ast \left({\rm{\Pi}}_{\rm{ca}=0}^{z}{f}_{\rm{ca}}\right),}\; \rm{otherwise}\end{array} \right. $$

where *Q*_mMSWp_ represents total mismanaged plastic waste from MSW of an urban agglomeration *i*, located in a river basin *j*. Each basin to which the urban or rural area corresponds to is chosen based on the location of the capital city. Previous studies (Lebreton et al., 2017; Schmidt et al., 2017) performed waste estimation per capita by using population density on a grid resolution (inhabitants per km^2^).

Each *f* in the equation represents a factor related to the amount of plastic waste that may be retained due to the action of natural and anthropogenic barriers and sinks. Urbanization and waste management systems are represented by the effect of their proximity ($\bar x$) towards either the coastline (*f*_cl_), their location inside an inter-basin (*f*_ci_), and the proximity to the main river in each watershed (*f*_cw_). The closer a human community is to either the coastline (*f*_cl_) or the main river on a watershed (*f*_cw_), the lower the retention and higher the amount of mismanaged waste that will enter the ocean (see Table 2).

**TABLE 2:** Proposed coefficients of waste dissipation

Factor	Characteristic	Coefficients of waste dissipation		
		Upper	Average	Lower
*f*_cl_	0–0.1 km	1.00	0.80	0.60
*f*_ci_	>0.1 km	0.20	0.10	0.05
*f*_cw_	0–0.1 km to main river	1.00	0.80	0.60
	>0.1–1 km to main river			
	Lower course	0.80	0.60	0.40
	Middle course	0.60	0.40	0.20
	Upper course	0.40	0.20	0.10
	>1–5 km to main river			
	Lower course	0.60	0.40	0.20
	Middle course	0.40	0.20	0.10
	Upper course	0.20	0.10	0.05
	>5 km to main river	0.05	0.01	0.00
*f*_rs_	Intermittent	1.00	0.80	0.60
	Perennial	1.00	1.00	1.00
*f*_ca_	Hydropower (run of the river)	0.80	0.40	0.20
	Hydropower (impoundment)	0.20	0.10	0.05
	Reservoirs, dams	0.80	0.40	0.20
	No barrier	1.00	1.00	1.00
*f*_sr_	Informal PET recovery	*r*_PET_*0.4	*r*_PET_*0.25	*r*_PET_*0.05

Abbreviations: *f*_cl_, coastline factor; *f*_ci_, inter-basin factor; *f*_cw_, closeness to main river factor; *f*_rs_, river seasonality factor; *f*_ca_, connection to an anthropogenic / natural barrier factor; *f*_sr_, scavengers’ recovery factor; r_PET_, fraction of PET bottles in waste stream.

Additionally, the effects of the characteristics of the river basins are quantified by assessing their seasonality (*f*_rs_). The effects of hydroelectric power plants, irrigation canals and drainage systems are represented by adding up the amount of these barriers displayed along the different river basins (*f*_ca_). Finally, the effects of informal waste recovery were also included (*f*_sr_). As discussed below, only the recovery of PET bottles was included to reduce the inherent uncertainty of this activity. It is important to note that factors range from 0 to 1. The closer to zero, the greater the retention of plastic waste.

Plastic pollution monitoring is needed to validate any numerical model at a local, regional, or global scale. Monitoring for long periods of time is needed to better understand the mobility of plastic waste towards the ocean (Lebreton et al., 2017). Waste dissipation coefficients proposed in this work should be further validated with field work.

The factors are described as follows:

- *f*_cl_: coastline factor and *f*_ci_: inter-basin factor. Inland sources of waste have been determined as the major contributors, thus, considering the riverine system as one of the biggest pathways for pWtO (Schmidt et al., 2017). However, coastal populations located beyond river catchment areas must also be considered as contributors of marine waste. Hence, we have also included two factors to analyze the influence of coastline population (*f*_cl_) and population located in inter-basins (*f*_ci_) which are not connected to a riverine system and are inside a watershed. Therefore, if a settlement is located within the first 100 m from the coastline, mMSW is highly likely to enter the ocean. In contrast, settlements located at a greater distance that are not connected to the riverine system (*f*_ci_) will experience greater retention inland with limited mobility towards the ocean.
- *f*_cw_: closeness to main river. Closeness of the settlement to the principal river in each watershed is assessed. The main river of a watershed will be the main pathway to transport plastic waste to the ocean; thus, the farther a settlement is from it, the more likely plastic waste will experience retention along its path. Distance to the coast also plays an important role, the further up in the river basin, the more likely it may be for waste to be trapped in different barriers. Thus, mismanaged waste located in the upper course of the basin will experience more retention than in the middle and the lower course of the basin (see Table 2). However, the possibility of waste being transported from greater distances should not be ruled out, considering the high level of uncertainty when modeling plastic transport in aquatic systems caused by the limited understanding of processes occurring during mobilization towards the ocean (Lebreton et al., 2017).
- *f*_rs_: river seasonality. This factor is related directly to the main river in the watershed where the capital city is located and depends on river characteristics: intermittent or perennial. We assume that intermittent rivers will halt waste transportation when dry, although other factors (e.g., wind, sedimentation) may continue the waste mobility cycle in the environment. Moreover, intermittent and perennial rivers may present important variations in their flow depending on seasonality, rainfall, or extreme weather events, in some cases overflowing and flooding the floodplains. Regardless of the frequency of waste entering the river floodplains, during the rainy season, river floodplains may be washed, transporting accumulated waste during the dry season towards the ocean. Thus, in most cases, river seasonality may only delay the mobility of waste. However, we assume that, considering a year-round evaluation, seasonality will still play a role in the retention of the plastic content of mismanaged MSW.
- *f*_ca_: connection to an anthropogenic or natural barrier (i.e., hydroelectric plant, reservoirs, canals, mangroves). This factor can be applied as many times as barriers are identified downstream the point of release. The product of sequence is used when more than one barrier is identified for the same settlement, being “z” equal to the number of barriers. As mentioned above, we are assuming a retention of up to 80% of waste for ROR hydroelectric plants with lower retention rates for the average and upper scenarios. Since information is lacking, the same retention rates for reservoirs and dams are considered. For impoundment facilities, however, higher retention rates may be expected, as reservoirs are part of the infrastructure.
- *f*_sr_2,: scavengers’ recovery. This factor quantifies the amount of plastic waste recuperated by informal pickers. Considering that it is not possible to estimate the exact location where waste is sorted and recuperated, a general percentage of waste removal is estimated. For this, the factors used by Peano et al. (2020) are applied. To reduce uncertainties, and following observations during field work, it is assumed that only PET bottles are being recuperated from the plastic fraction. Since this equation does not differentiate between plastic polymers, the percentage of plastic waste retrieved is calculated considering the average content of PET plastics in MSW streams. Three scenarios were assumed, considering a recuperation of 60%, 75%, and 95% of all PET bottles discarded and mismanaged (see Supporting Information S2, Table S1). This factor depends on the ratio of PET bottles in the waste stream (*r*_PET_), which is geographically specific.

For estimates performed using Equation (1), we propose the development of three scenarios considering an upper, lower, and average scenario, reflecting minimum, maximum, and average percentages of plastic waste retention following each type of barrier encountered. The upper scenario considers conservative retention capacities of the barriers identified, whereas the lower one estimates a higher retention ratio (see Table 2). Coefficients of waste dissipation are created, as stated before, to illustrate the effects of natural and anthropogenic barriers during waste transportation. However, uncertainty remains that will be mitigated through more detailed sampling and modeling in the future.

Even though wind and natural disasters have been identified as potential boosters or barriers during the displacement of waste towards the ocean, these were excluded from the equation. On the one hand, light-weighted MSW items incorrectly disposed of in open dumps are subject to be transported by wind and may, subsequently, reach a river basin or the ocean (Lebreton et al., 2017). However, the quantification of wind effects on waste transport was out of the scope of the current study. On the other hand, we consider that natural disasters should be assessed separately, since their consequences may differ based on intensity, location, and extension. Nevertheless, the evaluation of these events is necessary and should be assessed in depth in future studies.

### Step 3: Application of the proposed methodology to the Region of Piura (case study)

**FIGURE 2:**
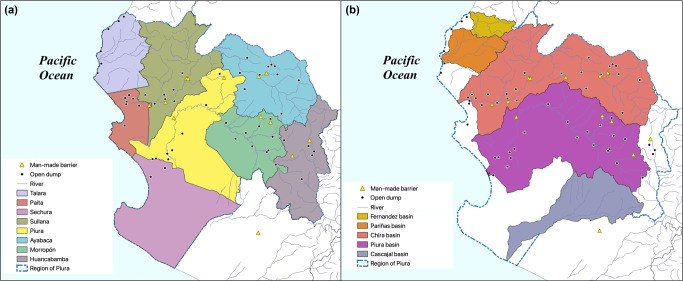
Study area. Black spots represent main open dumpsters located in the region. Yellow triangles represent identified manmade barriers. (a) The eight provinces inside the Region of Piura and (b) its watersheds

The Region of Piura (see Figure 2) is the second most populated department of Peru, with almost 1.9 million people. Although it is a coastal region, considering that half of the population is located inland translates into a high retention of the plastic content of mismanaged MSW due to geographical barriers (see Supporting Information S1, Section S1 for a detailed description of the region). However, most cities are situated next to riverbeds, thus increasing the likelihood of waste entering waterbodies.

The main rivers present relevant water flows during the summer months, with relatively low flows the rest of the year. Three out of five basins are classified as perennial, whereas the other two are intermittent basins, reaching the ocean mostly during the rainy season, minimizing the transport of mismanaged plastic during the dry season (see Figure 1). Settlements located in inter-basins (i.e., areas located between basins) are assumed to have higher inland retention, as they are mostly not connected to the riverine system. Also, different manmade barriers can be observed in the watersheds (e.g., 11 hydroelectric plants), although most barriers are located in the upper part of the region (see Figure 2), affecting a relatively small percentage of settlements.

When applying the methodology, for year 2018, 14,118 metric tons of pWtO were estimated on average, 7,883 metric tons for the lower scenario, and 26,049 metric tons for the upper scenario (see Table 3). These values translate into a per capita rate range from 4.2 to 13.9 kg plastic/year with an average of 7.5 kg plastic/year. Provinces located further from the ocean, in the basins of the rivers Chira and Piura, and with strong presence of hydropower plants, show the largest retention rates (see Supporting Information S1, Figure S2a). In contrast, coastal provinces (i.e., Talara and Paita) have the highest pWtO rates per person. These results represent modeling performed for the year 2018; however, in the period 2019–2021 four landfills were built to dispose of MSW in the cities of Sullana, Sechura, Paita, and Talara. Assuming *ceteris paribus* conditions but including these landfills into the calculations, a substantial reduction is observed, with a range of 2.2 to 8.8 kg/person/year and an average of 4.5 kg/person/year of mismanaged pWtO (see Supporting Information S1, Figure S2b).

**TABLE 3:** Estimation of plastic waste-to-ocean generation in the Region of Piura (Peru) based on mismanaged municipal solid waste for the year 2018

Province	Population	Total MSW(t/y)	Plastic fraction(t/y)	Formally recovered(t/y)	Totalplastic mismanaged(t/y)	pWtO			% Retention		
						Lower(t/y)	Average (t/y)	Upper(t/y)	Lower	Average	Upper
Ayabaca	127,735	26,290	2961	59	2902	0	2	35	100	100	99
Huancabamba	58,496	14,483	1631	33	1598	0	4	59	100	100	96
Morropón	167,461	46,819	5273	105	5167	80	426	1760	98	92	66
Paita	136,708	30,941	3485	70	3415	1596	2218	2848	53	35	17
Piura	828,343	211,295	23,797	476	23,321	3139	6651	12,184	87	71	48
Sechura	81,612	14,073	1585	32	1553	364	663	1061	77	57	32
Sullana	324,116	78,675	8861	177	8683	126	742	3854	99	91	56
Talara	146,248	40,579	4570	91	4479	2577	3411	4248	42	24	5
**Total**	1,870,719	463,155	52,162	1043	51,119	7883	14118	26,049	85	72	49

Abbreviations: BCS, best-case scenario; MSW, municipal solid waste; pWtO, plastic waste-to-ocean; WCS, worst case scenario.

Jambeck et al. (2015) estimated an average of 14.1 kg/person/year of plastic waste reaching the ocean in Peru, similar to the estimated upper scenario in the current study, but higher than the values obtained for the average and lower scenario. However, we expect Peruvian average plastic release values to be lower than the ones computed for Piura.  The current study considers regional data, avoiding the use of average data for upper middle-income countries. Additionally, we have incorporated the effects of natural and anthropogenic barriers along the transportation of plastic waste from MSW towards the ocean, which justifies the lower amounts estimated for marine waste in the average and lower scenarios. We hypothesize that the global estimation provided by Jambeck et al. (2015) may overestimate releases to the ocean in coastal environments where a compound of physical barriers retain waste from being released into the ocean, even in regions or nations with poor waste management systems.

## CONCLUSIONS

Identifying natural and anthropogenic barriers provides a more accurate picture of river dynamics and other potential sinks when transporting plastic waste towards the ocean. In fact, assuming a uniform behavior and transportation of waste in rivers may constitute an oversimplification that could provide erroneous estimations. While most studies related to marine litter have focused on phenomena occurring in coastlines and oceans, this study shifts the focus to include the interconnection of river basins, which have been identified as important precursors of marine litter accumulation, and the ocean. Along the Peruvian coast, where differing behaviors are observed throughout, the evaluation of the different river basins and catchment areas as possible final sinks for mismanaged plastic waste from MSW origin is necessary. This situation is related to the numerous natural and artificial boosters and barriers that may facilitate or interrupt the flow of waste towards the ocean.

The methodology proposed develops more accurate material flow analysis related to waste management and transportation of mismanaged plastic waste along different compartments, namely in the Global South, where research on the topic has been less prolific. We hypothesize that current worldwide global estimations may overestimate pWtO releases in coastal environments where a compound of physical barriers retains plastic waste from being released. In this sense, we argue that the use of this methodology may help in regionalizing plastic waste dissipation into the environment.

Despite the advantages of this regionalized approach, further research must be undergone to understand certain dynamics. For instance, wind transportation or extreme events (i.e., natural disasters such as mudslides or ENSO) will have an important impact on waste transportation. Similarly, further research regarding the dissipation rates avoided through the abovementioned barriers should be analyzed in further depth through sampling techniques.

Finally, increasing attention has been given to the effects of pWtO, leaving behind other ecosystems where debris are also becoming ubiquitous and abundant. Thus, there is also a need to consider the destination of mismanaged MSW in sinks other than the ocean (e.g., river basins or agricultural fields), which become relevant in countries with poor waste management systems. Thus, researchers and decision makers should not only focus on developing ocean cleaning strategies but increasing the scope of their analysis to deal with in-land strategies as well, that as shown in this article is connected with the former.

## Supplementary Information

**Supporting information S1**: This supporting information provides an in depth description of the case study. Additionally, it includes detailed tables of field work and the classification of river basins in the Peruvian Pacific Basin according to their seasonality. Finally, figures related to natural barriers and a graphical representation of plastic waste-to-ocean per capita of the case study are included.

**Supporting information S2**: This supporting information provides a table of plastic waste-to-ocean estimation for the Region of Piura, Peru.

## Data Availability

The data that support the findings of this study are available in the supporting information of this article. Additional data is also available from the corresponding author upon reasonable request.
