# Supplementary material for: Developing a methodology to quantify mismanaged plastic waste entering the ocean in coastal countries
Source: J Ind Ecol. 2022 Dec 16;26(6):2108–22. doi: 10.1111/jiec.13349 (PMC13098952; doi:10.1111/jiec.13349)
Supplement: Supplementary file 1 — Supporting information S1: This supporting information provides an in depth description of the case study. Additionally, it includes detailed tables of field work and the classification of river basins in the Peruvian Pacific Basin according to their seasonality. Finally, figures related to natural barriers and a graphical representation of plastic waste-to-ocean per capita of the case study are included. [file 44498_2022_2606022_MOESM1_ESM.docx]

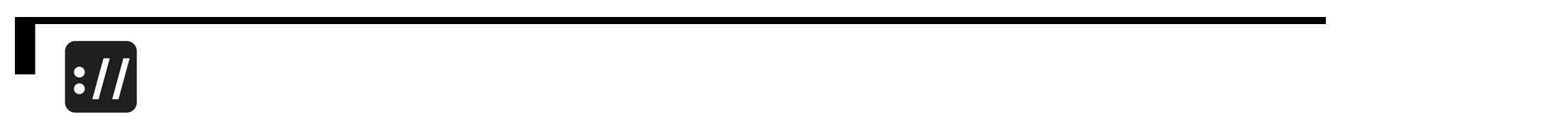


SUPPORTING INFORMATION FOR:

Ita-Nagy, D., Vázquez-Rowe, I. & Kahhat, R. (2022) Developing a methodology to quantify mismanaged plastic waste entering the ocean in coastal countries. *Journal of Industrial Ecology.*

**
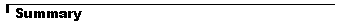
**

This supporting information provides an in depth description of the case study. Additionally, it includes detailed tables of field work and the classification of river draining basins in the Peruvian Pacific Basin according to their seasonality. Finally, figures related to natural barriers and a graphical representation of plastic waste-to-ocean per capita of the case study are included.


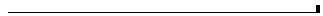


**Supplementary Text:**

**Section S1:** Application of the proposed methodology to the Region of Piura

The Region of Piura, located in northwestern Peru, is the second most populated department of Peru, with almost 1.9 million people and a total area of 35,892 km^2^. The capital city, also named Piura, is located in the middle basin of the river Piura, concentrating 44% of the total population and is relatively distant (ca. 108 km) from the coastline (INEI, 2017). The region is divided in eight provinces (see Figure 2a) and includes five watersheds in its territory (see Figure 2b). The reference year to estimate pWtO was 2018. MSW management in the region is poorly developed, with 60 open dumpsters identified, some located in the proximity of riverbeds (OEFA, 2021). The evaluation of the watersheds reveals that, besides years with an extreme ENSO event, a set of barriers may be responsible for retaining part of the mismanaged MSW that may reach the rivers along the basin.

The province of Talara, composed by six districts, is located mainly along the coastline. With a population of almost 150’000 people, and without an adequate landfill to dispose their solid waste, the likelihood of littering and waste entering the ocean is quite high, represented by the high f_cl_ factor. On the contrary, the district of Colan, in Paita, and the district of El Alto, in Talara, are both located further from the coastline (>0.1 km) on an inter-basin, far away from a waterbody. In these cases, mismanaged waste is most likely to stay inland and pollute either desertic areas or valleys.

Besides these locations, the rest are placed in one of the five basins in the region. For these districts, their closeness to the coast becomes less important than their closeness to the main river that drains into the ocean. The higher a community is located in the watershed (upper, middle or low course) and the further it is from the main river, the more waste will be retained or removed from the basin, represented by low f_cw_ factors. Contrarily, the lower it is located on the watershed and the closer to the main river, there will be higher chances for waste to be mobilized towards the ocean (higher f_cw_ factors).

Different man-made barriers can be observed in the watersheds, including 11 hydroelectric plants, as well as reservoirs and dams, used to diversify water towards agricultural lands. All hydroelectric plants are run-of-the-river, which translate in high retention of plastic waste coming upstream from these barriers. However, most barriers are located in the upper part of the region (see Figure 2), affecting a relatively small percentage of settlements (see Table S1).

Focusing back on the behavior of the watersheds, their seasonality will also affect the movement of waste towards the ocean. In this case, we evaluated the closest river for each settlement and their seasonality. However, it is important to mention that, since we are considering a period of one year, the rainy season will mobilize most waste retained during dry season. Thus, the upper value for either a perennial or intermittent river, in terms of retention, is assumed zero (no retention). However, considering river dredging activities can occur during dry season and may remove accumulated waste, we have considered retention values in the average and lower scenarios.

Finally, as mentioned in the main text, we included the influence of scavengers’ recovery of plastic waste along the path towards the ocean. Considering the high uncertainty of this activity, we have only assumed a recuperation of PET bottles, as it was observed and corroborated during field work. PET bottles represent the 4.61% of the total of plastic waste generated (SIGERSOL, 2018), and the amount retrieved from this fraction was calculated using the Plastic Leak Project (Peano et al., 2020) removal estimations of valuable plastic materials.

**Supplementary Tables:**

**Table S1:** Plastic waste-to-ocean estimation for the Region of Piura, Peru

(See Excel file)

**Table S2:** Field work performed in the basin of river Piura and other sites throughout the country, as part of the evaluation of Peruvian Pacific basin

| **Date** | **Location** | **Area description** | **Findings** |
| --- | --- | --- | --- |
| 08/12/2019 | Cercado de Lima, Lima, Lima | Rimac river in Lima | Part of a Waste Collection Day activity. A section of the river basin was cleaned up. Presence of mismanaged solid waste along the riverbanks, including MSW and non-MSW (e.g., tires, WEEE). |
| 17/02/20 | Morropón, Morropón, Piura | Open dump in Morropón | Little to no coverage, presence of abundant vectors and waste combustion, mostly MSW. Presence of waste pickers in the area, mostly collection of PET, mix plastic, aluminium and iron. |
| 17/02/20 | La Matanza, Morropón, Piura | Open dump in La Matanza | Small open dump, presence of herd of sheep, mostly MSW. Presence of waste pickers in the area, mostly collection of PET and aluminium, extensive burning of waste. |
| 17/02/20 | Piura, Piura, Piura | Water irrigation channels | Located throughout the city of Piura. Presence of light weighted waste transported downstream. |
| 18/02/20 | Tambogrande, Piura, Piura | Open dump in Tambogrande | Located next to an irrigation channel. Waste accumulation in piles along the area. Presence of waste pickers and waste combustion.  Waste collection center located outside of open dump, trade of all types of recyclable waste. |
| 19/02/20 | Curamori, Piura, Piura | Open dump, water irrigation channels and WWTP in Curamori | Relatively controlled open dump, with sand coverage of waste. Almost no presence of vectors.  Implementation of a composting plant for organic waste (pilot).  Retention of waste along the floodgates and surrounding areas. Collapsed WWTP. |
| 19/02/20 | Sechura, Sechura, Piura | Piura river, irrigation channels and Chulliyachi mangrove | Presence of solid waste along the river and channels. Accumulation of waste along the riverbanks, mainly lightweight and buoyant items. |
| 20/02/20 | Castilla, Piura, Piura | Open dump in Piura | Main open dump of the capital city of the region, Piura. Presence of abundant vectors. Mainly MSW.  Important presence of waste pickers. Waste collection center located outside of open dump, trade of all types of recyclable waste. |
| 15/01/21 | Casma, Casma, Ancash | Port and river in Casma and beach in Tortugas | Waste transported by river towards the ocean. Accumulation of waste was also observed at the north of the Casma bay (*Rincón de Piños*). In Tortugas, a combination of fishing gear, aquaculture waste and MSW were found on the beach and surrounding areas. |
| 28/05/21 | Pisco, Pisco, Ica | River Pisco and coastal area. Open dumps at Humay and Pisco | Identification of construction and demolition waste from the earthquake in Pisco (2007) located in multiple open dumps.  Identification of waste disposal along the river basin from human settlements and farm workers.  Open dumps are located inside sand dunes. Waste collection was identified inside.  Identification of waste accumulation along the coast and at the discharge of a water canal. |
| 28/05/21 | Paracas, Pisco, Ica | Open dump in Paracas | The open dump is located inside sand dunes. Waste pickers were identified inside. |
| 29/05/21 | Mala, Cañete, Lima | Open dump in Mala, and mouth of river Mala | Disposal of waste into the Mala river basin was observed. Waste was identified along the river.  Waste was identified along the river mouth on the beach. Waste corresponds to typical organic materials carried by a river (branches) and MSW, either transported by the river or from beachgoers.  A non-identified open dump was located next to the *C.P. 27 de Diciembre*. |
| 29/05/21 | Asia, Cañete, Lima | Open dump in Asia and non-identified open dump | Open dump with relatively high coverage. A second open dump was located nearby. |
| 26/01/22 | Barranca, Barranca, Lima | Beach | Identification of waste accumulation along the coast. |
| 26/01/22 | Supe, Barranca, Lima | Beach and dock | Identification of waste accumulation along the coast and related to artisanal fishing. |
| 26/01/22 | Huacho, Huaura, Lima | Beach and dock | Identification of waste accumulation along the coast and related to artisanal fishing. |
| 25/02/22 | Lima, Lima, Peru | Four beaches located in the north and south of Lima | Visual identification of waste and information gathering related to beachgoers behaviour |
| 07/03/22  &  18/03/22 | Ventanilla, Lima, Peru | Chillón river | Mouth of the river with high presence of plastic waste and other materials |
| 27/03/22  &  24/04/22 | Coishco, Santa, Ancash | Beach and fish meal plant | Identification of waste accumulation along the coast. |
| 27/03/22  &  24/04/22 | Chimbote, Santa, Ancash | Beach, dock and fish meal plant | Identification of waste accumulation along the coast. |
| 27/03/22  &  24/04/22 | Casma, Casma, Ancash | Beach, dock and open dump | Identification of waste accumulation along the coast and related to artisanal fishing and aquiculture. |
| 27/03/22  &  24/04/22 | Huarmey, Huarmey, Ancash | Beaches, dock, fish meal plant and Huarmey river | Identification of waste accumulation along the coast, at the discharge of the river mouth and related to artisanal fishing. |
| 27/03/22  &  24/04/22 | Huaura, Huaura, Lima | Beach, fish meal plant, open dump and Huaura river | Identification of waste accumulation along the coast and at the discharge of the river mouth. |
| 29/03/22 | Cercado de Lima, Lima, Lima | Rimac river | Identification of waste accumulation along the coast and at the discharge of the river mouth. |
| 24/04/22 | Guadalupito, Virú, La Libertad | Beach, Santa river | Identification of waste accumulation along the coast and at the discharge of the river mouth. |

**Table S3:** Classification of river draining basins in the Peruvian Pacific Basin according to their seasonality.

| **Main watershed** | | | **Pacific** | **Classification according to seasonality** |
| --- | --- | --- | --- | --- |
| **Order** | **WMA** | **Code** | **Basin name** |  |
| 62 | WMA V: JEQUETEPEQUE - ZARUMILLA | 13952 | Zarumilla | Perennial |
| 61 |  | 1394 | Tumbes | Perennial |
| 60 |  | 13936 | Bocapán | Perennial |
| 59 |  | 13934 | Quebrada Seca | Perennial |
| 58 |  | 13932 | Fernández | Perennial |
| 57 |  | 1392 | Pariñas | Perennial |
| 56 |  | 138 | Chira | Perennial |
| 55 |  | 1378 | Piura | Intermittent |
| 54 |  | 13778 | Cascajal | Intermittent |
| 53 |  | 137774 | Olmos | Intermittent |
| 52 |  | 137772 | Motupe | Perennial |
| 51 |  | 13776 | Chancay-Lambayeque | Intermittent |
| 50 |  | 137754 | Zaña | Perennial |
| 49 |  | 137752 | Chamán | Perennial |
| 48 |  | 13774 | Jequetepeque | Perennial |
| 47 | WMA IV: HUARMEY - CHICAMA | 13772 | Chicama | Intermittent |
| 46 |  | 137716 | Moche | Perennial |
| 45 |  | 137714 | Virú | Intermittent |
| 44 |  | 137712 | Huamansaña | Intermittent |
| 43 |  | 1376 | Santa | Perennial |
| 42 |  | 1375992 | Lacramarca | Intermittent |
| 41 |  | 137598 | Nepeña | Intermittent |
| 40 |  | 137596 | Casma | Intermittent |
| 39 |  | 1375952 | Culebras | Perennial |
| 38 |  | 137594 | Huarmey | Perennial |
| 37 | WMA III: CAÑETE - FORTALEZA | 137592 | Fortaleza | Perennial |
| 36 |  | 13758 | Pativilca | Perennial |
| 35 |  | 137572 | Supe | Intermittent |
| 34 |  | 13756 | Huaura | Perennial |
| 33 |  | 137558 | Chancay - Huaral | Perennial |
| 32 |  | 137556 | Chillón | Perennial |
| 31 |  | 137554 | Rímac | Perennial |
| 30 |  | 1375534 | Lurín | Perennial |
| 29 |  | 1375532 | Chilca | Intermittent |
| 28 |  | 137552 | Mala | Perennial |
| 27 |  | 1375512 | Omas | Perennial |
| 26 |  | 13754 | Cañete | Perennial |
| 25 |  | 137534 | Topará | Perennial |
| 24 | WMA II: CHAPARRA - CHINCHA | 137532 | San Juan | Perennial |
| 23 |  | 13752 | Pisco | Perennial |
| 22 |  | 1374 | Ica | Perennial |
| 21 |  | 1372 | Grande | Perennial |
| 20 |  | 13718 | Acarí | Perennial |
| 19 |  | 13716 | Yauca | Perennial |
| 18 |  | 137158 | Honda | Intermittent |
| 17 |  | 137156 | Chala | Intermittent |
| 16 |  | 137154 | Cháparra | Perennial |
| 15 | WMA I: CAPLINA - OCOÑA | 137152 | Choclón | Intermittent |
| 14 |  | 13714 | Atico | Perennial |
| 13 |  | 13712 | Pescadores - Caraveli | Intermittent |
| 12 |  | 136 | Ocoña | Perennial |
| 11 |  | 134 | Camaná | Perennial |
| 10 |  | 132 | Quilca - Vitor - Chili | Perennial |
| 9 |  | 1318 | Tambo | Perennial |
| 8 |  | 13178 | Honda | Intermittent |
| 7 |  | 13172 | Ilo - Moquegua | Perennial |
| 6 |  | 1316 | Locumba | Perennial |
| 5 |  | 13158 | Sama | Perennial |
| 4 |  | 13156 | Caplina | Intermittent |
| 3 |  | 13154 | Hospicio | Perennial |
| 2 |  | 13152 | De la Concordia | Perennial |
| 1 |  | 1314 | Lluta | Perennial |
|  |  |  |  |  |
| WMA= Water Managment Authorities (AAA, in spanish) | | | |  |

**Supplementary Figures**

**Figure S1:** Natural barriers observed in Pisco, Peru in May 2021.


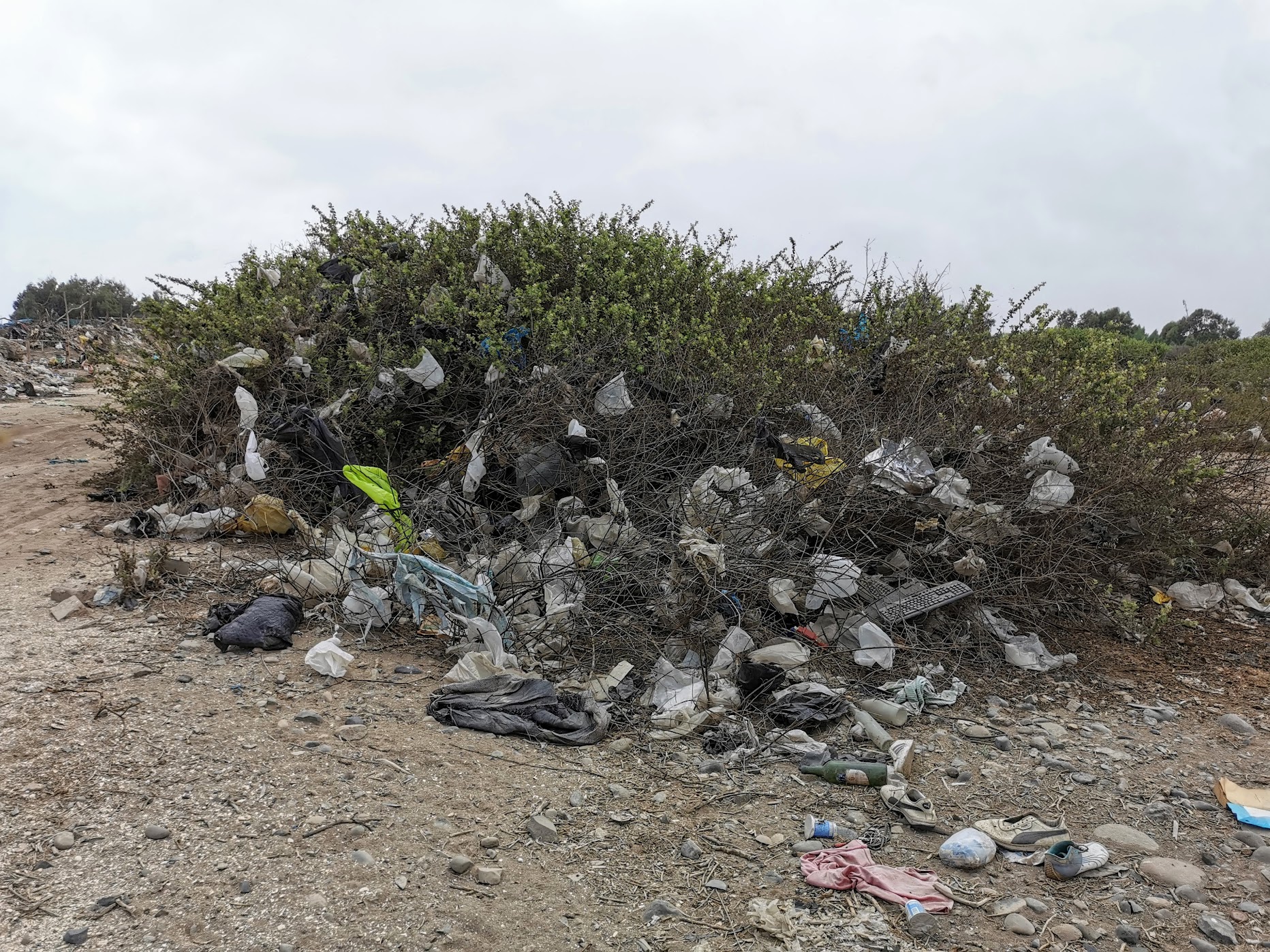

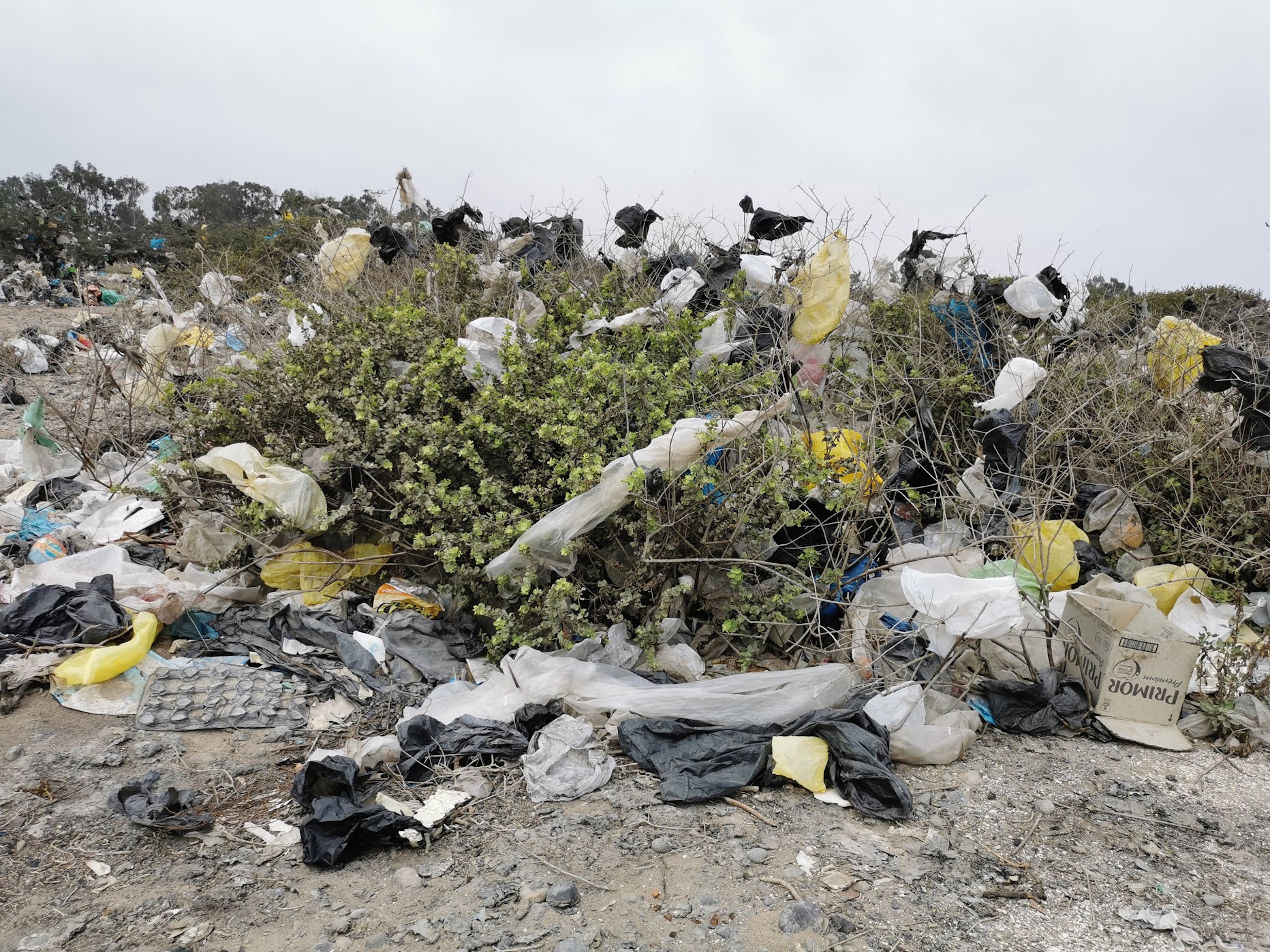


**Figure S2:** Graphical representation of plastic waste-to-ocean (pWtO) amounts in the region of Piura per province and per capita for the lower and upper scenarios. Fig. S2a depicts the pWtO amounts in the year 2018, prior to the construction of four landfills. Fig. S2b depicts the pWtO amount assuming ceteris paribus and including the four landfills built in the period 2019-2021.
